# Types of necroinflammation, the effect of cell death modalities on sterile inflammation

**DOI:** 10.1038/s41419-022-04883-w

**Published:** 2022-05-02

**Authors:** Anett Mázló, Viktória Jenei, Sára Burai, Tamás Molnár, Attila Bácsi, Gábor Koncz

**Affiliations:** Department of Immunology, Faculty of Medicine, University of Debrecen, Egyetem square 1, Debrecen, 4032 Hungary

**Keywords:** Necroptosis, Inflammasome

## Abstract

Distinct types of immune responses are activated by infections, which cause the development of type I, II, or III inflammation, regulated by Th1, Th2, Th17 helper T cells and ILC1, ILC2 and ILC3 cells, respectively. While the classification of immune responses to different groups of pathogens is widely accepted, subtypes of the immune response elicited by sterile inflammation have not yet been detailed. Necroinflammation is associated with the release of damage-associated molecular patterns (DAMP) from dying cells. In this review, we present that the distinct molecular mechanisms activated during apoptosis, necroptosis, pyroptosis, and ferroptosis lead to the release of different patterns of DAMPs and their suppressors, SAMPs. We summarize the currently available data on how regulated cell death pathways and released DAMPs and SAMPs direct the differentiation of T helper and ILC cells. Understanding the subtypes of necroinflammation can be crucial in developing strategies for the treatment of sterile inflammatory diseases caused by cell death processes.

## Facts

- Significantly different molecular mechanisms regulate the release of DAMPs in each regulated cell death process.
- Different DAMPs, cytokines and chemokines are released during various regulated cell death processes.
- Cell death processes have different effects on the differentiation of Th and ILC cell subpopulations.

## Open questions

- Are there precise subtypes of necroinflammation?
- Can the types of necroinflammation be related to the Th1, Th2, Th17 classification system?
- Do specific regulated cell death pathways trigger different types of necroinflammation?

## Introduction

The response of sentinel immune cells, primarily macrophages, dendritic cells and mast cells, initiates the processes leading to the classical signs of inflammation. The pattern recognition receptors (PRRs) of the initiator cells are activated by pathogen-associated molecular patterns (PAMPs), and also by damage-associated molecular patterns (DAMPs). Sterile inflammation in the absence of pathogens can be induced by DAMPs released from the cells or formed in the interstitial space. The most common mechanism of sterile inflammation is necroinflammation, where DAMP production associated with dysregulated cell death is the cause of the process [1]. However, at the same time, during cell death, various suppressing/inhibiting DAMPs (SAMPs, iDAMPs, or also termed special pro-resolving mediators SPMs) could be released, including molecules such as prostaglandin E2 [2] (PGE2), resolvins, protectins or maresins, and lipoxins [3] that contribute to inflammation-resolving pathways [4]. Sterile inflammation, especially in its chronic form, could be the cause of many different diseases, including but not limited to various forms of neurodegenerative disorders, skin- and intestinal tract-related diseases, obesity, atherosclerosis, hepatitis, pancreatitis and also chronic low-grade inflammation in the elderly [5] (Table 1). However, the exact cause and mechanism of the cell death process in most inflammatory diseases is not yet clear.

**Table 1:** Examples of inflammatory diseases in which apoptosis, necroptosis, pyroptosis or ferroptosis are involved.

Tissue/cell death	Apoptosis	Necroptosis	Pyroptosis	Ferroptosis
Neurodegenerative disorders	Alzheimer's disease, Parkinson's disease [108, 109], Huntington's disease [109]	Multiple sclerosis^,^ Alzheimer’s disease, amyotrophic lateral sclerosis [14]	Multiple sclerosis, Alzheimer's disease, Parkinson's disease, Huntington's disease [110]	Alzheimer's disease, Parkinson's disease, Huntington's disease [31]
Metabolic disorders	Obesity, insulin resistance, type-2 diabetes [111]	Crohn’s disease, inflammatory bowel disease [14]	Type 1–2 diabetes, inflammatory bowel disease [112]	Diabetes mellitus [113]
Liver diseases	Nonalcoholic steatohepatitis cirrhosis [111]	Alcoholic liver disease, nonalcoholic fatty liver disease [14]	Alcoholic hepatitis, nonalcoholic fatty liver disease [112]	Nonalcoholic fatty liver and alcoholic liver diseases, hemochromatosis, drug-induced liver injury [114]
Autoimmune diseases	Systemic lupus erythematosus, rheumatoid arthritis [109]	Rheumatoid arthritis, autoimmune arthritis [115]	Sjogren's syndrome, rheumatoid arthritis, systemic lupus erythematosus [112]	Systemic lupus erythematosus [116]
Cardiovascular diseases	Atherosclerosis, cardiovascular diseases [108, 109]	Chronic heart failure [14]	Atherosclerosis, ischemic heart disease, myocardial infarction [111, 117]	Atherosclerosis [114]
Skin diseases	Systemic lupus erythematosus [108]	Lichen planus, systemic lupus erythematosus [14], psoriasis [115]	Psoriasis [118]	Keratinocyte death [119]
Pulmonary diseases	Chronic obstructive pulmonary disease [108] acute respiratory distress syndrome [120]	Chronic obstructive pulmonary disease [115]	Acute lung injury [111]	Chronic obstructive pulmonary disease [113]
Dental disorders	ND	Periodontitis [14]	Gingivitis [121]	ND
Cancer	Breast carcinoma, colorectal cancer and many other tumors [122]	Pancreatic cancer [14] breast cancer [123–125]	Liver, gastric tissues, uterine, cervical and breast cancers [111], melanoma,lung cancer, colorectal cancer [126]	Hepatocellular carcinoma [114]
Disorders in the reproductive tract	Preeclampsia [127]	Reproductive senescence, early menopause [128]	Preeclampsia, preterm birth [111], endometriosis, infertility, reproductive senescence [129]	Preeclampsia [130]
Kidneys	Nephropathy, acute kidney injury [131]	Acute kidney injury, autosomal dominant polycystic kidney disease [14]	Acute kidney injury [113]	Inflammation and tissue injury in kidney [31] acute kidney injury [115]

Over the past few years, new cell death processes have been described and classified as regulated cell death modalities that can be characterized by well-defined, unique, tightly regulated signaling pathways [6]. These cell death processes differ in the morphology and enzyme activity of dying cells, and their impact on the outcome of innate and adaptive responses. Most of these regulated cell death mechanisms have been classified as necrotic, proinflammatory cell death stimulating innate immune reactions. Activation of adaptive immunity, especially cytotoxic T cells by immunogenic cell death has also been described [7]. However, the interaction between different inflammatory cell death processes, necroptosis, pyroptosis, and ferroptosis with different types of helper T cells has not been studied.

The main characteristic profile of infections, i.e. the presence of intracellular, extracellular or parasitic pathogens, determines the three main types of immune responses that are supervised by Th1, Th17, Th2 cells. These branches of the immune response differ functionally and also in the composition of reactive cells. As well as the costimulatory molecule profile and cytokine milieu, the presence and proportion of DAMPs and SAMPs may be critical in the polarization of the T cell response.

We attempted to identify a link between the different cell death pathways and their effect on the polarisation of the immune response. In this review, we study the immunological outcome of necroinflammatory cell death pathways one by one. We compare molecules released during apoptosis, necroptosis, pyroptosis, ferroptosis, and determine immunological decision-making on how these cell death pathways regulate Th1, Th2, Th17, and regulator T cell-directed immune responses through secreted DAMP and SAMP profiles.

## Directions of immune response according to helper T cell subtypes

After intracellular, extracellular and parasitic infections, various cells and humoral factors specific to each type of immune response are activated. According to these pathogen groups, subtypes of the immune response can be classified, represented by groups of helper T cells (Th1, Th2, and Th17), cells that direct each immune arm principally by producing an appropriate cytokine panel. Regulatory T cells complete this picture as a general suppressor of the three kinds of the immune response.

In the last decade, innate lymphoid cells (ILC) have been described as regulators of the immune response and classified into ILC1, ILC2, and ILC3 subtypes according to their differentiation and function. As part of innate immunity, these cells respond early to infections and regulate the immune response principally by their cytokine production.

**Fig. 1:**
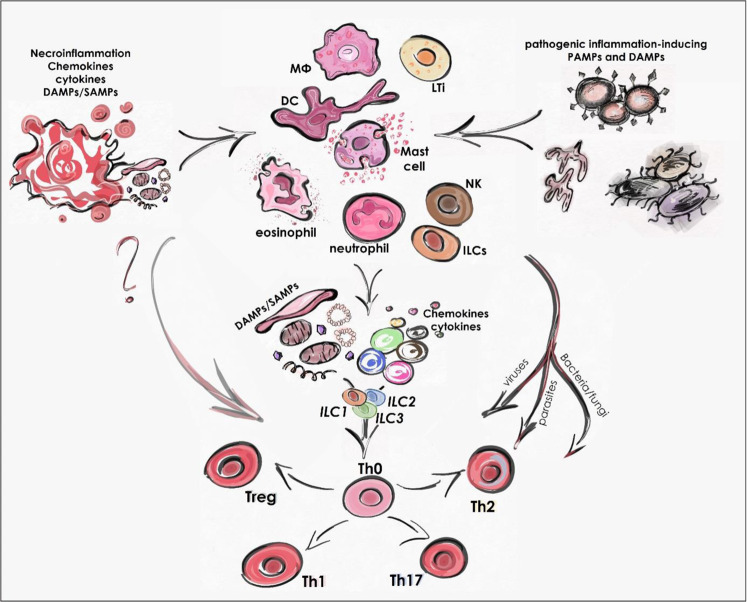
Sterile inflammation in parallel with pathogen-induced immune response. Distinct classes of pathogens induce different immune responses, commonly known as type 1, 2, and 3 immunity, resulting in the activation of different T helper cell subsets. While pathogen-induced immune responses can be classified into different subclasses, called type 1, 2, and 3 inflammation, DAMP-induced necroinflammation is not yet linked to this partitioning system. Necroinflammation can be classified as an independent type of inflammation that can be well distinguished from the other three major classes, or necroinflammation may also have subtypes. If it can be divided into subtypes, these subtypes can be compatible with subclasses of pathogen-induced processes, or can be different from them, inducing unique types of immune reactions.

Tissue environmental factors, particularly the cytokine profile of sentinel immune cells, are critical in determining the polarization of Th and ILC cells. Th1/ILC1 differentiation of the immune response is initiated by the presence of type I and type II interferons, while IL-12 and IL-18 promote the production of interferon-γ (IFN-γ). Th2/ILC2 differentiation is mediated, among others, by IL-4, IL-13, IL-33; Th17/ILC3 by IL-1, IL-6, IL-23 and peripheral Tregs by TGFβ and IL-2 production [8]. As stimulant factors, these cytokine motifs determine the direction of the immune response and the types of inflammation (Fig. 1).

## Different mechanisms in cell death modalities are responsible for the release of DAMPs

Various cell death pathways have become known in recent decades. Here we focus only on the molecular background of DAMP release during apoptosis, necroptosis, pyroptosis, and ferroptosis [9], but comprehensive review abouts cell death pathways are available [6, 10].

Apoptosis, the most common process of cell death, is a highly regulated, active mechanism mediated by the caspase cascade. It can be divided into internal (also known as mitochondrial) or external apoptotic pathways, depending on the inducing stimuli. Activation of the caspase cascade ultimately leads to cell elimination. During the process, apoptotic bodies are formed by plasma membrane blebbing, preventing the release of intracellular content [11]. Membrane-coated apoptotic bodies with a diameter of 50–5000 nm carry the intracellular components and are completely engulfed by nearby cells and/or professional phagocytes in a process called efferocytosis [12]. Due to its general immunosuppressive and tolerogenic effect, apoptosis is traditionally accepted as physiological cell death. Although apoptosis is considered an immunologically silent process, it has been shown that in addition to apoptotic bodies, immunomodulatory microvesicles and exosomes can also be released from apoptotic cells. The ratio of the amount of apoptotic cells to the efferocytic capacity also influences the immunological outcome of apoptosis, as insufficient engulfment results in disintegration of apoptotic bodies and thus leads to DAMP release. During apoptosis, a number of kinases and transcription factors are inactivated by caspases, thereby inhibiting the production and secretion of inflammatory cytokines [13].

Necroptosis is an inflammatory form of regulated cell death that can be triggered by perturbations in either the external or internal microenvironment [14]. The formation of the RIPK1/RIPK3 necrosome eventually leads to the phosphorylation and activation of MLKL molecules [15]. Activated MLKL molecules are oligomerized and translocated to the cell membrane where they form cation-selective ion channels. These membrane pores are characterized by an estimated internal diameter of 4 nm [16]. MLKLs also activate cation channels, such as the transient receptor potential melastatin-related 7 (TRPM7) [17]. The generated ionic disturbance results in the swelling of cells and organelles, leading to the rupture of the plasma membrane and thus, the release of DAMPs. Once MLKLs bind to the membrane, a number of pathways can be initiated against necroptosis that facilitate cell survival and also affect the release of DAMPs. The endosomal sorting complexes required for transport III machinery (ESCRT-III) mediates the shedding of plasma membrane [18], while the flotillin-mediated endocytosis and ALIX-syntenin-1-mediated exocytosis all act to remove the membrane-bound activate MLKLs [19]. Enzymes of the disintegrin and metalloprotease (ADAM) family are also activated by phosphorylated MLKLs in adherent cells. These proteins induce the shedding of various cell surface proteins (e.g. adhesion molecules, receptors, growth factors, and cytokines), contributing to the release of DAMP in the early stages of necroptosis [20]. Nuclear factor-κB (NF-κB)-induced transcription may occur during necroptosis, allowing active cytokine production by dying cells [21], and thus co-released DAMPs and cytokines may determine the polarization of inflammation.

Classically, the recognition of PAMPs and DAMPs by PRRs as a primary signal sensitizes cells to inflammasome-formation and pyroptosis by inducing the activation of inflammatory caspases (caspase-1/-4/-5/-11) through the activation of multiple inflammatory complexes. Activation of these specific caspases leads to the cleavage of members of the gasdermin (GSDM) superfamily, which then are polymerized and form pores in the plasma membrane [22, 23]. GSDM pores are sufficiently large, the inner diameters ranging from 10 to 18 nm, allowing the release of low-molecular-weight cellular contents [24]. Caspase-1-mediated cleavage of IL-1 family cytokines (IL-1β and IL-18) creates their active forms and allows their secretion [23]. Unlike in necroptosis, the GSDMD-N formed pores are non-selective, so intracellular osmolality changes are less intense during pyroptosis than necroptosis. Consequently, cell death can occur independently of cell lysis in pyroptosis [24, 25]. However, like necroptosis, activation of pyroptosis can also elicit membrane repair pathways to rescue cells through the ESCRT-III machine to remove membranes damaged by GSDM pores [26]. As a result of these events, the cell survives, but inflammatory mediators may still be released through the pores before GSDMs are removed from the membrane [25, 26]. Classically, the recognition of PAMPs and DAMPs by PRRs as a primary signal sensitizes cells to inflammasome-formation and pyroptosis. Certain signaling pathways, such as extracellular signal-regulated kinase (ERK), an activator of signal transduction and transcription 3 (STAT3), and phosphatidylinositol 3 kinase (PI-3K) may be active due to active PRR signaling [27]. These signaling pathways together with DAMP release, IL-1, IL-18 and also chemokine production act in a unique pattern to amplify the inflammation during pyroptosis [9].

It has become clear that plasma membrane rupture is not a passive but a highly regulated event. Surprisingly, oligomerization of the Ninjurin-1 (NINJ1) protein also regulates the membrane permeability and the release of DAMPs in apoptosis, necrosis and pyroptosis, but not in necroptosis [28]. These results link the mechanisms of DAMP release during tolerogenic apoptosis and highly inflammatory pyroptosis, while sharply separating these processes from the immunological outcome of necroptotic cell death.

**Fig. 2:**
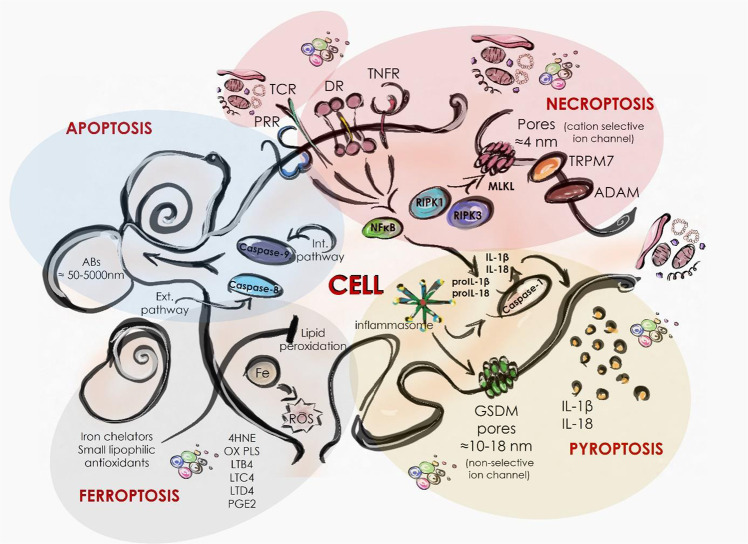
Comparison of the main processes leading to DAMP release in apoptosis, necroptosis, pyroptosis and ferroptosis. During apoptosis, caspase-regulated events lead to the wrapping of intracellular components into apoptotic bodies. Subsequent efferocytosis prevents the release of DAMPs into the extracellular space. Necrosome induction results in the activation of cation-selective ion channels, leading to cell lysis due to osmotic shock. Pyroptosis is characterized by the secretion of IL-1 and IL-18 cytokines by inflammasome activation and the formation of voluminous non-selective pores formed by GSDMs. In ferroptosis, oxidative perturbations accumulate toxic lipid peroxides that ultimately cause the DAMP release. PRR Pattern Recognition Receptor DR death receptor, GSDM gasdermin, ADAM a disintegrin and metalloproteinase, TRPM7 Transient receptor potential cation channel, subfamily M, member 7, 4HNE 4-Hydroxynonenal, PGE2 Prostaglandin E_2,_ OX PLS oxidized glycerophospholipids, LTB4 Leukotriene B4, LTC4 Leukotriene C4, LTD4 Leukotriene D4, RIPK1 Receptor-interacting serine/threonine-protein kinase, MLKL Mixed lineage kinase domain-like pseudokinase.

Ferroptosis, an iron-dependent form of necrotic cell death, is mediated by the accumulation of lipid peroxidation, and can be prevented by iron chelators and small lipophilic antioxidants [29]. Cells undergoing ferroptosis show necrosis-like morphological changes: cell enlargement, swelling of organelles, membrane rupture [30]. Although not yet fully elucidated, it is known that DAMPs are also released during ferroptosis. The ESCRT-III machinery counteracts various forms of regulated necrosis, including ferroptosis [31]. Endoplasmic reticulum stress plays a potential role in this mechanism, as calcium signals are required to membrane repair [32]. In addition to DAMP release, ferroptosis interacts with the RAS/MAPK/ERK pathway, allowing for a different pattern of cytokine production than in pyroptosis [33]. Ferroptosis also directly activates arachidonic acid metabolism and eicosanoid biosynthesis, resulting in the release of lipid oxidation products (4HNE, oxPLs, LTB4, LTC4, LTD4 and PGE2) [30]. Thus, factors secreted in ferroptosis can directly affect inflammation, similar to IL-1 released in pyroptosis, while activation of innate immune cells through DAMP/SAMP production also indirectly regulates inflammation (Fig. 2).

## The role of DAMPs and their suppressors in the regulation of inflammation

DAMPs are typically derived from dying cells, although DAMPs can be released from living cells exposed to severe stress or by damage of extracellular matrix proteins [34]. Secretory lysosomes and exosomes also have been published as carriers of DAMPs during their active release such as HMGB1, ATP, histones, HSPs, RNAs and DNA [9].

DAMP release during cell death does not automatically initiate inflammation, only the activation of tissue-resident sensory cells (primarily macrophages and DCs) results in the production of inflammatory mediators. Sterile inflammation should be considered as a multi-step process [1]. Various, frequently unknown stimuli induce cell death [2], inflammatory forms of cell death results in DAMP or SAMP secretion, which is detected mostly by innate immune cells [3]. Activated cells produce cytokines, inflammatory and pro-resolving mediators, among them newly appeared DAMPs and SAMPs [4, 35, 36]. Some of these mediators have an autocrine effect on innate immune cells and some can also induce cell death [4]. In this complex process, DAMPs and SAMPs of dying cells and mediators released by innate immune cells altogether determine whether inflammation can progress into a chronic reaction, or the resolving of inflammation and tissue regeneration are induced. The exact individual role of most DAMPs in inflammation is not yet clear, but it can be hypothesized that simultaneous detection of different DAMPs may significantly amplify the danger signal, since it was described that after the activation of TLR receptors the interaction of several receptors may elicit a synergistic effect [37]. With this in mind, the diversity, amount, and half-life of DAMP/SAMP released from dying cells and the balance of mediator secreted efferocytes both influence subsequent reactions [38].

## Different DAMPs are released during various regulated cell death processes

As we detailed above, cell death processes are characterized by different signal transduction pathways, execution mechanisms and various secretory routes for DAMP release. Necroinflammation induced by dying cells can lead to the release of different qualities and quantities of DAMPs/SAMPs. The characteristic secretory DAMP pattern for each cell death mode has not yet been systematically studied, and only a few comparative studies are available where DAMPs secreted by different cell death pathways are compared. In Table 2, we collected the available data on which cell death modality leads to which DAMP production. The cell death-specific classification of individual DAMPs is complicated by the facts that secreted DAMPs/SAMPs may change over time during death processes, that cell death procedures can be progressive or even parallel events [36], and different subtypes of a particular cell death pathway may modify the immunological outcome of the processes [10, 39].

**Table 2:** DAMPs and SAMPs released in different cell death forms.

	Apoptosis	Necroptosis	Pyroptosis	Ferroptosis
DAMPs	• HMGB1 [10] • ATP [9] • DNA [9] • IL-1α [132] • IL-33 [120] • Histones [10] • RNA [9] • S100 proteins [133] • HSPs [134] • Uric acid [135] • mtDNA [136] • EMAP II [137] • Low molecular weight nucleotides [10]	• HMGB1 [132] • ATP [10] • DNA [9] • IL-1α [132] • IL-33 [10] • Histones [10] • RNA [9] • S100A9 [10] • HSPs [9] • Uric acid [138] • mtDNA [10] • Long genomic DNA [138] • CIRP [9] • Cyclophilin A [139] • Spliceosome-associated Protein 130 [140]	• HMGB1 [132] • ATP [9] • DNA [9] • IL-1α [132] • IL-1β [132] • IL-18 [132] • IL-33 [132] • mtDNA [132] • eCIRP [141] • ASC specks [10]	• HMGB1 [9] • ATP [142] • DNA [10] • mtDNA [143] • Oxidized phospholipids [30] • Malondialdehyde [144]
SAMPs	• Annexin A1 [145] • Resolvin D1 [146] • Resolvin E1 [147] • Lipoxin A4 [148] • Prostaglandin-E2 [149] • Maresin [35] • Protectin [150]	• Annexin A1 [151] • Resolvin D1 [85] • Resolvin E1 [85] • Resolvin D5 [85] • Lipoxin A4 [85] • Maresin 1 [85]		• Resolvin E1 [152]

*HMGB1* high mobility group box 1, *CIRP* cold-inducible RNA-binding protein, *EMAP* II endothelial-monocyte-activating polypeptide II.

It is well known that when the intensity of apoptosis exceeds efferocytotic capacity, apoptosis turns into secondary necrosis. Similarly, early and late ferroptotic cells have been shown to differ in their ability to induce DC maturation in the co-culture assays [40]. Pyroptosis is a cellular response to the detection of DAMPs and PAMPs, so the release of DAMP following any cell death mode may induce pyroptosis as the second wave of cell death [41]. Apoptosis may follow necroptosis over time, for example in the cerebral ischemia-reperfusion stroke, which aggravates neuronal inflammation [42]. When cells suffer programmed necrosis, neighboring cells may become sensitive to necrotic cell death leading to synchronized regulated necrosis. These processes can simultaneously activate different programmed necrotic pathways in the affected tissue [43]. Dozens of articles also provide data that a blockage of a cell death route activates other death pathways, resulting in the desirous elimination of the cell, but occasionally inducing different immunological outcomes. For example, ferroptosis and necroptosis act in a coordinated manner: if the activity of one pathway decreases, that of the other improves to compensate it [42].

Symbolic molecules of cell death pathways have been described to regulate or initiate other cell death pathways. For example, caspase-3 and caspase-8 have been described as initiators of pyroptosis [22] and vice versa, caspase-1 was also indicated as the regulator of effector caspases [44]. While caspase-8 is a well-known inhibitor of necroptosis, caspase-9 has been published to be necessary for the death receptor and PRR-induced necroptosis [45]. Acetylated p53 and the pro-apoptotic Bid protein have been shown to contribute to ferroptosis [46, 47]. Key molecules of necroptosis RIPK1, RIPK3 were indicated as apoptosis regulators and RIPK1, RIPK3 and MLKL are also involved in pyroptosis [48]. Expression of TAK1 [49], RIPK1 [50] and oxygen radicals [51] seems to affect all the mentioned cell death pathways, and ESCRT-III machinery can modify DAMP release in all of these necrotic cell death subroutines [52]. Molecular entanglement of cell death pathways may result in PANoptosis, bringing together the components of apoptosis necroptosis and pyroptosis into a complex [53]. This contemporaneous function of cell death pathways has been observed, especially after infections, but also in auto-inflammatory and metabolic disorders [54]. In addition, it is still unclear whether a particular type of cell death occurs through different signaling pathways, results in the same DAMP/SAMP production, or leads to different immune responses (Table 3).

**Table 3:** Pros and cons of different forms of necroinflammation.

Pros	Cons
Cell death modalities differ in the mechanism of DAMP release	Each cell death process can be activated by multiple signaling pathways
Different DAMPs are released during cell death modalities	Timely different DAMPs can be released in the same cell death pathway
Critical DAMPS of the Th differentiation, such as IL-1, IL-33, SAMPs seems to be produced dominantly upon specific cell death forms.	Cell death pathways can work simultaneously
Different posttranslational modifications of DAMPs are activated upon different cell death pathways	Cell death processes can be sequential events, activating each other.

## Immunological decision-making I: how do the different DAMP molecules organize the type of immune response?

Some DAMPs are ubiquitously expressed molecules (e.g. actin, ATP, HSP) that allow any cell to alert the immune system. Most DAMPs can induce DC maturation and migration into the lymph nodes (LNs) and consequently stimulate naïve T cell activation. Accordingly, DAMPs can trigger the sensitization phase of allergic disorders (Th2 response), but they also contribute to graft rejection (Th1 response) [55]. However, DAMPs generally activate the innate immune response, and consequently, naïve T cells, based on current knowledge, only a few DAMPs appear to be dominant in determining the T helper cell polarization. The extracellular appearance of actin, HMGB1, and/or type I interferons is key for Th1 differentiation, IL-1 production promotes Th17 differentiation, IL-33 is known as a critical cytokine in the Th2 response, and finally, SAMPs promote polarization to Treg cells. It should be noted that some DAMP stimulates the Th0 profile, such as HSP60 with concomitant induction of IL-10 and INF-γ production [56].

### DAMPs in the induction of Th1 response

For APCs, phagocytosis of intracellular antigens available in dying cells induces MHC-I-associated expression, allowing efficient activation of CTLs specific for intracellular contamination. In contrast, phagocytosis of extracellular pathogens should not lead to MHC-I-mediated antigen presentation to prevent CTLs from killing the effective APCs. Accordingly, the engulfment of dead cells by DCs leads to a different mode of antigen presentation than in the case of phagocyted extracellular pathogens. Uptake only of dead human cells induces cross-presentation, exceptionally allowing the simultaneous presentation of phagocyted antigens by MHC-I and MHC-II, activating both CTLs and Th1 cells. Primarily, cells that die due to immunogenic cell death (ICD) are effective in activating CTLs [7]. ICD is not a specific mode of regulated cell death, but a variety of cell death processes, any of which result in a cytotoxic T cell response.

Some DAMPs are highlighted as necessary actors in ICD: [7], HMGB1, ATP, calreticulin, annexin A1, DNA, HSP and actins, but the precise role of ICD-associated DAMPs remains to be fully elucidated. Some of these DAMPs (e.g. ATP) facilitate the recruitment of APCs into the environment of dying cells, some promote the interaction between dying/dead cells and APCs (e.g. ANXA1), increase the efficiency of phagocytosis of dying cells (e.g. CALR, HSPs, F-actin), assist APC in cross-presentation (e.g. ATP, HMGB1, type I IFN and TFAM, HSPs) [7]. DAMPs released during immunogenic cell death must not only activate immature DCs to deliver antigens to LNs (adjuvant effect), but must also specifically mark the source of the phagocyted antigen, indicating that it is derived from dead cells, which may increase cross-presentation [57]. One such DAMP is extracellular F-actin, as its receptor on CLeC9A (DNGR1) on DCs is involved in the recognition of necrotic, but not early apoptotic cells and enables cross-presentation of dead cell-derived antigens to CD8 + T cells [58, 59].

In addition to contributing to cross-presentation, some DAMPs have been mentioned to directly regulate Th cell differentiation to Th1 cells. HMGB1 is essential for DC maturation, migration to lymphoid tissues and also for polarization of naïve T cells to functional Th1 cells. HMGB1 polarizes the Th1 response via the enhanced secretion of TNF-a IL-6 and IL-12 [60]. In systemic lupus erythematosus, histone H1 was mentioned to constitute the induction of Th1 response as T cell autoantigens [61]. The inflammasome-activated IL-18 acts synergistically with the typical Th1 cytokine, IL-12, and induces the production of IFN-γ [62]. Caspase-1 is responsible for converting IL-18, but also IL-1β into mature forms. IL-1β undoubtedly has a role in the induction of delayed-type hypersensitivity responses [63, 64], and it also induces M1 macrophage polarization [65], but its direct role in the dominant induction of the Th1 response is quite questionable. Regarding the interaction between DAMPs and Th1-supporting ILC cells, two DAMPs, namely HSP70 and HMGB1, have been shown to result in higher IFNγ production in ILC1 and NK cells under oxidative stress in vitiligo [66].

### DAMPs in the induction of Th2 response

It is widely accepted that IL-33 promotes Th2 responses. It has been implicated in the pathogenesis of Th2-related diseases, asthma and atopic dermatitis [67], and IL-33 has also been shown to exert protective effects upon parasitic infection [68]. Treatment with recombinant IL-33 promoted a Th2 (IL-4, IL-13 and IL-9) and Treg response, whereas it suppressed Th1- and Th17-type cytokine expression (IFNγ, TNFα and IL-6) [69]. IL-33 has been identified as necroptotic DAMP due to its increased expression in necroptotic epidermal keratinocytes [70], thus linking necroptosis to the type 2 immune response [71]. Other cell death pathways are less likely to induce IL-33 production since it is cleaved by caspases during apoptosis. Some authors have also indicated caspase-1-driven inactivation of this cytokine, but in contrast, IL-33 release following pyroptosis has been also mentioned [72]. Among the DAMPs, in addition to IL-33, S100A8/A9 has been mentioned to shift the Th1/Th2 balance towards Th2 and thereby amplify the allergic cascade in food allergy [73].

IL-33 is involved not only in the differentiation of Th2, but also in the activation of ILC2 cells. Tissue-resident ILC2s in the lung are activated by inhaled allergens via epithelial-derived IL-33. Furthermore, intranasal IL-33 administration increased the numbers of ILC2s in the lung, also in peripheral blood and in the liver [74].

### DAMPs in the induction of the Th17 response

There is no doubt that IL-1 is the most important DAMP in the regulation of Th17 cells. It is required for Th17 differentiation as well as for the function of effector Th17 cells in synergy with IL-6 and IL-23 [75, 76]. S100 proteins have also been shown to promote the development of Th17 cells, as activation of monocytes by the S100 protein results in differentiation of the Th17 subpopulation in patients with acute graft-versus-host disease (GvHD) [77].

Th17 response could be promoted by ILC3 cells. Cooperation of CD39 and CD73 receptors converts inflammatory extracellular ATP to tolerogenic adenosine. Some human ILC3 cells co-express CD39 and CD73, especially after IL-1β stimulation. In addition, the extracellular ATP-activated ILC3 cells produce IL-22, a typical cytokine of the Th17 response [78]. Additionally, increased amounts of ILC3 cells are associated with upregulated levels of IL-1β and aggravates inflammatory arthritis in mice lacking phagocytic NADPH oxidase. Accordingly, treatment with IL-1 antagonists effectively lowered the proportions of IL-17A producing ILC3 cells in Ncf1-/- arthritic mice and ameliorated the joint inflammation [79].

### Regulatory role of SAMPs and DAMPs in immune tolerance

Programmed apoptotic cell death has historically been considered a tolerogenic pathway. Indeed, after apoptosis, various tolerance-inducing mechanisms can be activated, including regulatory T cell differentiation and blocking the polarization of other T helper phenotypes. Apoptotic cells can themselves produce immunomodulatory factors such as IL-10 and TGF-β1. In addition, upon contact with apoptotic cells, activated monocytes switch from a pro-inflammatory to an anti-inflammatory state, not only to suppress immune and inflammatory responses, but also to promote the clearance of apoptotic cells [80]. Both apoptotic cells and macrophages interacting with apoptotic cells produce pro-resolving mediators or SAMPs [81]. However, the microenvironment can also regulate SAMP production, as M1 and M2 macrophages possess distinct SAMP profiles [82]. PGE2 appears to be a critical mediator of apoptosis-induced immune tolerance, as it promotes the production of SAMPs, namely resolvins, protectins, lipoxins and maresins [2, 35]. The released SAMPs stimulate tissue regeneration in many ways and lead to tolerance, including stimulation of tolerogenic cytokine production, enforcing the differentiation of tolerogenic cell populations, such as M2 macrophages and regulatory T cells, and by blocking of immunogenic reactions [35, 83, 84]. Much less data has been published on SAMP production in non-apoptotic forms of cell death (Table 2), but increased SPM biosynthesis has been shown following successful clearance of necroptotic cells in atherosclerotic plaques [85].

In addition to direct induction of immunotolerance and Treg cell differentiation and activation, PGE2, and SAMPs actively block the differentiation and function of other T cell and ILC subpopulations. PGE2 selectively inhibits Th1 activation, but in addition to generating Tregs, it also promotes T cell polarization toward the highly plastic Th17 phenotype [86]. RvD1, RvD2, RvE1, and maresin all inhibit IL-17 secretion by Th17 cells, suggesting that SPMs may use common intracellular signaling pathways that regulate Th17 function [84, 87]. D-series resolvins (resolvin D1 and resolvin D2) and maresin also reduce cytokine production by Th1 cells. Moreover, these SPMs prevented not only cytokine production, but the differentiation of naïve CD4 + T cells into Th1 and Th17 by down-regulating their signature transcription factors [88]. PGE2 had a profound inhibitory effect on IL-33-induced ILC2 expansion and on the production of critical Th2-related cytokines IL-5 and IL-13 in vitro [89]. Lipoxins also inhibit ILC-2 cells to decrease airway inflammation [90]. ILC2s express receptors for pro-resolving mediators, consequently, LXA_4_ and MaR1 can potently inhibit the release of ILC2-derived pro-inflammatory cytokines [83, 90].

Immune tolerance induced by apoptosis is the result not only of SAMP production, but also of active inhibition of DAMPs critical for Th1, Th2 and Th17 differentiation. Caspase-mediated cleavage of DNA or mtDNA limits stimulation of DNA sensors prohibiting interferon production [91]. During apoptotic cell death, HMGB1 is oxidized, reversing its proinflammatory function toward tolerogenicity [92]. Caspase-dependent proteolysis of IL-33 has been published to dramatically attenuate IL-33 bioactivity [93]. GSDMD has also been identified as a caspase-3 target and its enzymatic cleavage leads to loss of function [94].

## Immunological decision-making II.: how do the different necrotic cell death pathways affect the type of immune response?

**Fig. 3:**
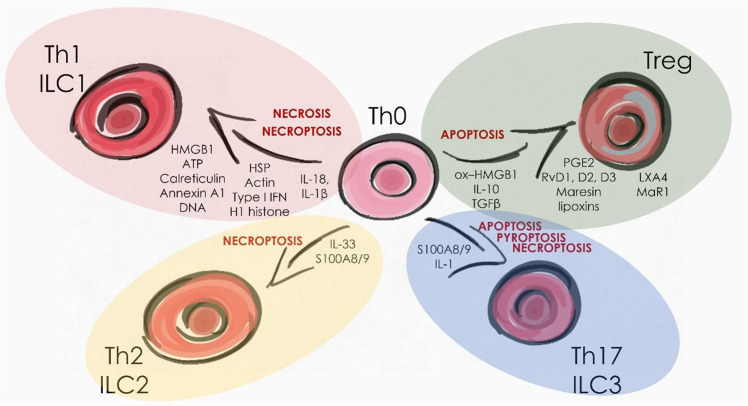
DAMP and SAMP molecules regulating Th cell differentiation. Types of cell death, DAMP and SAMP molecules directly associated with T helper cell subpopulations based on literature data. HMGB1 High mobility group box 1, PGE2 Prostaglandin E_2_, TGFβ Transforming growth factor-beta.

The polarization of naïve and effector T cells is also strongly influenced by the soluble mediators appearing in their microenvironment. Different DAMPs, SAMPs and cytokines are produced during each cell death modality. In this section, we have collected how different cell death pathways may affect helper T cell and ILC polarization, which determines the type of inflammation (Fig. 3).

### Cell death in the induction of Th1 response

High levels of TLR4 in necrotizing enterocolitis (NEC) activate necroptosis of intestinal epithelial cells, contributing to local inflammation that may have been prevented by the use of Necrostatin-1, a necroptosis inhibitor [95]. Significant increases in the level of IL-6, IL-17 and TNFalpha have been mentioned in NEC, confirming the role of cell death in Th1-and Th17-related inflammation. In hyperinflammatory tumors, the microenvironment is generally rich in iron, which, by inducing oxidative stress and cell death on T cells and B cells, contributes to the progression of cancer. Iron chelating agents can be used to treat the iron overload, which has been demonstrated to increase the Th1 response [96, 97]. Thus, induction of ferroptosis in immune-excluded tumors may provide all the benefits of “immunogenic cell death” without the disadvantages of immunosuppression.

### Cell death in the induction of Th2 response

Cadmium toxic pollutant reduces the production of IFNγ and increases the levels of IL-4, IL-6 and IL-10, thereby weakening the Th1 response but enhancing the activity of Th2 cells [98]. Cadmium induces necroptosis with increased expression of RIPK1, RIPK3 and MLKL and cadmium-induced inflammation is inhibited by necrostatin-1 in a porcine model. Further enhancing the effect of necroptosis on Th2 polarization, ILC2s have been shown to produce IL-4 prior to any other cell types following doxorubicin (DOX)-induced myocardial necroptosis [99].

### Cell death in the induction of Th17 response

Necroptosis may also support the effector activity of Th17 cells following recognition of fungi. A special case of this is when necroptosis of macrophages in a process called metaphorosis can lead to calcineurin-dependent lateral transfer of *A. fumigates* to live macrophages to downregulate fungal germination [100, 101]. Selenium deficiency induced accelerated cell necroptosis in IPEC-J2 cells, resulting in increased expression of IL-1β, IL-6, IL-7 and IL-17, while the expression of IFNγ, IL-10 and IL-4 is down-regulated indicating a Th17 polarization of helper T cells [100]. Intestinal epithelial cell necroptosis, which results in the recruitment and activation of ILC3s and IL-22 production, also enhances Th17 responses [102]. IL-18 released by pyroptotic cells is involved in promoting the IL-17 production of Th17 cells in osteoporosis. In this process, pyroptotic activation of NLRP3 contributes to bone damage also through activation of T lymphocytes [103]. In the intestinal lamina propria, in the presence of commensal microbes and dietary antigens, a sensitive, highly plastic balance of Th17 and Treg cells is established. The uptake of apoptotic cells by DCs induces their IL-6 and TGFβ production, leading to the differentiation of Th17 cells from naïve CD4 + lymphocytes [104]. However, in this microenvironment, the presence of microbes in apoptotic cells is highly likely, and accordingly TLR2 activation precedes the production of Th17 cytokines.

### Cell death in the induction of regulatory T (Treg) cell response

Apoptotic cells are easily recognized and absorbed by phagocytes, leading to their anti-inflammatory activity. In this process efferocytes secrete anti-inflammatory cytokines, like IL-10 and TGFβ, but less of inflammatory cytokines such as TNFα, IL-1β and IL-12. The presence of apoptotic cells induces the differentiation of naïve T cells into Treg cells, and also suppresses the activity of effector T lymphocytes [105]. In addition, the uptake of apoptotic cells results in a decrease in MHC-II expression on the surface of professional antigen-presenting cells (APCs). Modification of MHC and co-stimulatory/inhibitory receptor expressions presumably plays a critical role in Th17 and Treg induction during DC-T cell interaction [106]. However, APC types can elicit different responses to apoptotic cells. Macrophages can induce mainly tolerogenic, while DCs can induce immunogenic responses. Uptake of apoptotic cells by phagocytes not only induces regulatory T cell differentiation, but also inhibits polarization of other T helper subtypes by inhibiting the production of IL-12, IFNγ, and Th17 cell-derived cytokines, IL-17 and IL-23 [107].

## Conclusions

Usually, the late diagnosis of diseases makes it difficult to conclude whether the abnormal immune inflammation is the cause or result of an illness. Sterile inflammation can be the cause of many diseases, so its possible subtypes, their association with Th subpopulations, should be better elucidated. Characterization of the immunological outcome of cell death can determine the points of translational intervention in the regulation of many forms of sterile inflammation.

## Supplementary information

Author contribution statement

checklist

## Data Availability

All data generated or analyzed during this study are included in this published article.
