# Supplementary material for: Types of necroinflammation, the effect of cell death modalities on sterile inflammation
Source: Cell Death Dis. 2022 May 2;13(5):423. doi: 10.1038/s41419-022-04883-w (PMC9061831; doi:10.1038/s41419-022-04883-w)
Supplement: Supplementary file 1 — Author contribution statement [file 41419_2022_4883_MOESM1_ESM.pdf]

UNIVERSITY of DEBRECEN

FACULTY OF MEDICINE
DEPARTMENT OF IMMUNOLOGY
H-4032 Debrecen, 1 Egyetem square
Phone: +3652/417-159, email: immunology.office@med.unideb.hu

Author Contribution Statement REF: CDDIS-22-0313RR

Manuscript entitled: Types of necroinflammation, the effect of cell death modalities on sterile inflammation

The authors confirm contribution to the paper as follows:

Conceptualization, A.M. and G.K.; Writing—Original Draft Preparation, V.J., A.M., S.B.,TM. and G.K Writing—Review and Editing, A.B., A.M. and G.K.; Visualization, A.M. and G.K.; Supervision, G.K. Funding Acquisition, A.B., and G.K. All authors have read and agreed to the published final version of the manuscript.

The authors hereby confirm that they have agreed to the submission of this version of the manuscript and they have no conflict of interest to declare.

Anett Mázló

Viktória Jenei

Sára Burai

Tamás Molnár

Attila Bácsi

Gábor Koncz
